# The impact of integrated hepatitis B virus DNA on oncogenesis and antiviral therapy

**DOI:** 10.1186/s40364-024-00611-y

**Published:** 2024-08-15

**Authors:** Mingming Zhang, Han Chen, Huan Liu, Hong Tang

**Affiliations:** 1 Center of Infectious Diseases, West China Hospital of Sichuan University, Chengdu, 610041 China; 2 Laboratory of Infectious and Liver Diseases, Institute of Infectious Diseases, West China Hospital of Sichuan University, Chengdu, 610041 China

**Keywords:** Hepatitis B virus, Virus DNA integration, Hepatocellular carcinoma, Antiviral therapy

## Abstract

The global burden of hepatitis B virus (HBV) infection remains high, with chronic hepatitis B (CHB) patients facing a significantly increased risk of developing cirrhosis and hepatocellular carcinoma (HCC). The ultimate objective of antiviral therapy is to achieve a sterilizing cure for HBV. This necessitates the elimination of intrahepatic covalently closed circular DNA (cccDNA) and the complete eradication of integrated HBV DNA. This review aims to summarize the oncogenetic role of HBV integration and the significance of clearing HBV integration in sterilizing cure. It specifically focuses on the molecular mechanisms through which HBV integration leads to HCC, including modulation of the expression of proto-oncogenes and tumor suppressor genes, induction of chromosomal instability, and expression of truncated mutant HBV proteins. The review also highlights the impact of antiviral therapy in reducing HBV integration and preventing HBV-related HCC. Additionally, the review offers insights into future objectives for the treatment of CHB. Current strategies for HBV DNA integration inhibition and elimination include mainly antiviral therapies, RNA interference and gene editing technologies. Overall, HBV integration deserves further investigation and can potentially serve as a biomarker for CHB and HBV-related HCC.

## Background

Chronic Hepatitis B (CHB) refers to a persistent liver infection caused by the hepatitis B virus (HBV) that lasts for more than 6 months [1]. Among these individuals with CHB, a staggering 820,000 cases experienced adverse outcomes such as liver failure, liver cirrhosis, HBV-related hepatocellular carcinoma (HCC), or other HBV-associated diseases. The risk of developing HCC in patients with CHB is significantly elevated, ranging from 10 to 100 times higher compared to non-infected individuals. Integrated HBV DNA refers to the incorporation of HBV DNA into the host cell’s genome, which is one of the important factors contributing to HBV-related carcinogenesis. HBV integration can induce genetic damage and chromosomal instability, leading to tumor progression via the activation of oncogenes or inactivation of tumor suppressor genes [2–4]. It may persist even after successful hepatitis B surface antigen (HBsAg) seroconversion, which can contribute to HBsAg re-positivity and increase the risk of developing HCC [5]. Remarkably, integrated HBV DNA is identified in around 85-90% of HCC cases associated with hepatitis B virus infection [6]. Targeting the HBV DNA integration process and eliminating integrated HBV DNA from the host genome becomes crucial in preventing the progression of chronic hepatitis B. The treatment of CHB now includes options such as nucleos(t)ide analogues (NAs) and interferon-alpha (IFN-α), which could effectively inhibit HBV replication and new integrations. While IFN is administered for a finite duration, NAs are typically prescribed for extended periods, often lifelong [1, 7, 8]. The primary objective of CHB treatment is to suppress HBV replication to the maximum extent possible. Hepatocyte inflammation and necrosis, liver fibrosis and hyperplasia can be attenuated by inhibiting HBV replication, thereby delaying and reducing the occurrence of severe complications such as liver failure, liver cirrhosis, and HCC [9, 10]. The types of CHB cure are categorized as functional cure (also known as clinical cure or immunological cure) and sterilizing cure (also known as virological cure). A “functional” cure was defined as sustained HBsAg loss and HBV DNA less than the lower limit of quantitation (LLOQ) 24 weeks off-treatment [11]. On the other hand, a “sterilizing” cure was defined as all traces of HBV infection would be eliminated, including cccDNA and integrated HBV DNA [11, 12]. For eligible patients, the pursuit of functional cure should be considered [7, 13, 14]. The sterilizing cure of HBV is to achieve sterilizing cure, which necessitates the elimination of intrahepatic cccDNA and the complete eradication of integrated HBV DNA. However, this remains a challenging feat due to the persistence of viral reservoirs, weak immune response, long-term medication requirements, variable treatment responses, and the presence of advanced liver disease [1, 15]. In animal models, the frequency of integration events is estimated to be approximately one in 10^3^-10^4^ hepatocytes [16, 17]. However, the precise mechanisms of integration remain unclear, necessitating further attention and investigation. This review aims to provide a comprehensive overview of HBV DNA integration, including its molecular mechanisms, detection methods, research models, oncogenetic roles in HCC, and potential treatment strategies for eliminating HBV DNA integration.

## HBV integration occurs during HBV replication

### The structure and life cycle of HBV DNA

**Fig. 1:**
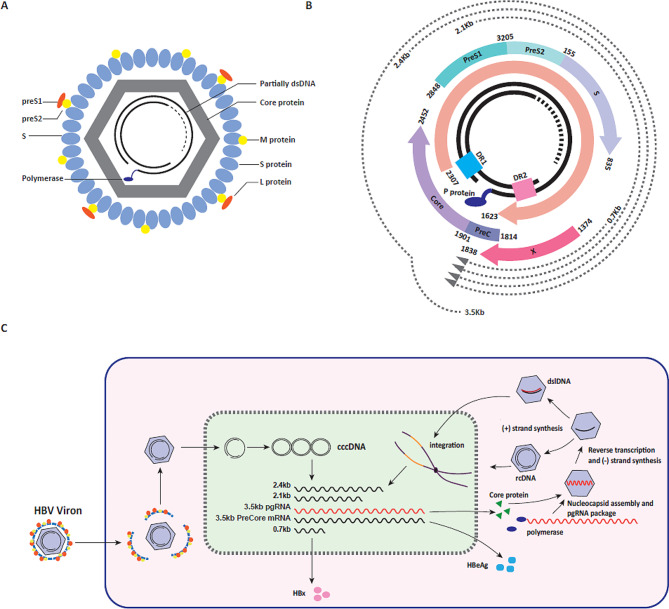
The structure of HBV DNA genome and HBV life cycle. **(A)**The schematic diagram of Dane particle. **(B)**The circular schematic diagram of genotype C HBV genome. **(C)** After HBV Dane particles entering hepatocytes, uncoating takes place and genome is released. RcDNA is repaired to form cccDNA which is transcribed to pgRNA and 3.5Kb/3.5Kb/2.4Kb/2.1Kb/0.7Kb transcripts, HBV RNA transcripts are translated into proteins such as HBeAg, Core protein and HBx protein. Polymerase binds to the pgRNA with the recruitment of Core protein to assemble nucleocapsid and package pgRNA, pgRNA serves as the template to reverse transcription synthesize the HBV minus(-)-strand DNA. Polymerase translocates accurately to synthesize the HBV plus(+) strand DNA. Polymerase translocates mistakenly results the synthesis of dslDNA and the integration of dslDNA into the host genome

The HBV is a DNA virus that belongs to the Hepadnaviridae family [1]. Dane particles are infectious virions characterized by a lipid membrane that encapsulates a nucleocapsid. The lipid membrane contains three HBsAg, which include large (L-HBs), middle (M-HBs), and small (S-HBs) forms. The nucleocapsid within the Dane particle is composed of hepatitis B core protein (HBc), viral polymerase (Pol), and the viral genome DNA. The HBc protein provides structural integrity to the nucleocapsid, while the viral polymerase is responsible for replicating the viral genome during the viral life cycle (Fig. 1A).

The viral genome of the HBV is a partially double-stranded DNA (dsDNA) structure. It consists of one complete coding minus(-) strand and one incomplete non-coding plus(+) strand. The viral genome contains four overlapping open reading frames (ORFs) that are responsible for encoding various viral proteins including: (1) HBV DNA polymerase (pol), which is involved in viral replication and synthesis of the viral genome. (2) HBsAg, which exists in three forms: Large, Medium, and Small. These antigens are crucial for viral attachment and entry into host cells. (3) HBV core antigen (HBcAg), providing structural integrity to the nucleocapsid of the virus. (4) HBV e antigen (HBeAg), whose exact function is not fully understood but is associated with immune tolerance and viral replication. (5) HBV X protein (HBx), a multifunctional protein involved in regulating viral replication, cell proliferation, and immune response modulation. Each of these viral proteins contributes to different aspects of the HBV life cycle, including viral replication, virion assembly, and immune evasion (Fig. 1B) [18].

After the HBV virion entering hepatocytes, the nucleocapsid is released into the cytoplasm and the relaxed circular DNA (rcDNA) enters the nucleus where it will convert into cccDNA [19–22]. The cccDNA then serves as a template for the synthesis of five transcripts (3.5Kb pregenomic RNA, 3.5Kb precore mRNA, 2.4Kb preS1 mRNA, 2.1Kb preS2/S mRNA, and 0.7Kb HBx mRNA), which are transcribed by host RNA polymerase II. Among these transcripts, the 3.5Kb pregenomic RNA (pgRNA) plays a crucial role in viral reverse transcription and replication processes. Following the utilization of pgRNA as a template, the minus(-) strand is synthesized, and subsequently, the synthesis of the plus(+) strand proceeds using the minus(-) strand as a template. This process gives rise to two products during plus(+) strand synthesis: partially circular rcDNA and double-stranded linear DNA (dslDNA). Rather than being encapsulated and secreted as virions, dslDNA has the potential to re-enter the nucleus and integrate into the human genome [5] (Fig. 1C).

### The procedure of HBV integration

During reverse transcription, the HBV DNA polymerase utilizes pgRNA as a template to transcribe the minus(-) strand DNA. This process involves the generation of DNA oligonucleotides (TGAA or GAA), which serve as primers for synthesizing the minus(-) strand. These primers are produced within a ε stem-loop structure located at the 3’ terminal of the pgRNA. During the process of extending the minus(-) strand DNA, most of the pgRNA undergoes degradation mediated by RNase H, except for the capped 5’ end of pgRNA. Remarkably, these undegraded RNA oligonucleotides play a vital role as primers for the synthesis of the plus(+) strand DNA. Among these fragments, one containing the direct repeat 1 (DR1) sequence acts as a primer for the synthesis of the plus(+) strand DNA [23–25]. Typically, this primer binds to the direct repeat 2 (DR2) region of the newly synthesized minus(-) strand DNA, which is complementary to the DR1 segment of the residual pgRNA. This binding guides the synthesis of plus(+) strand DNA, resulting in the formation of partially circular rcDNA (90–95%). However, if the initiation of the plus(+) strand synthesis occurs in situ without binding to the DR2 region (in-situ priming), the synthesis of double-stranded dslDNA takes place (5–10%) [26]. This small probability of primer translocation failure might be due to the mutation of DR2 region which causing reduced complementarity between DR2 region and RNA primer [27]. Another study suggests that cis-acting elements mutants in HBV genome are related to the proportion of dslDNA generation [28, 29].

**Fig. 2:**
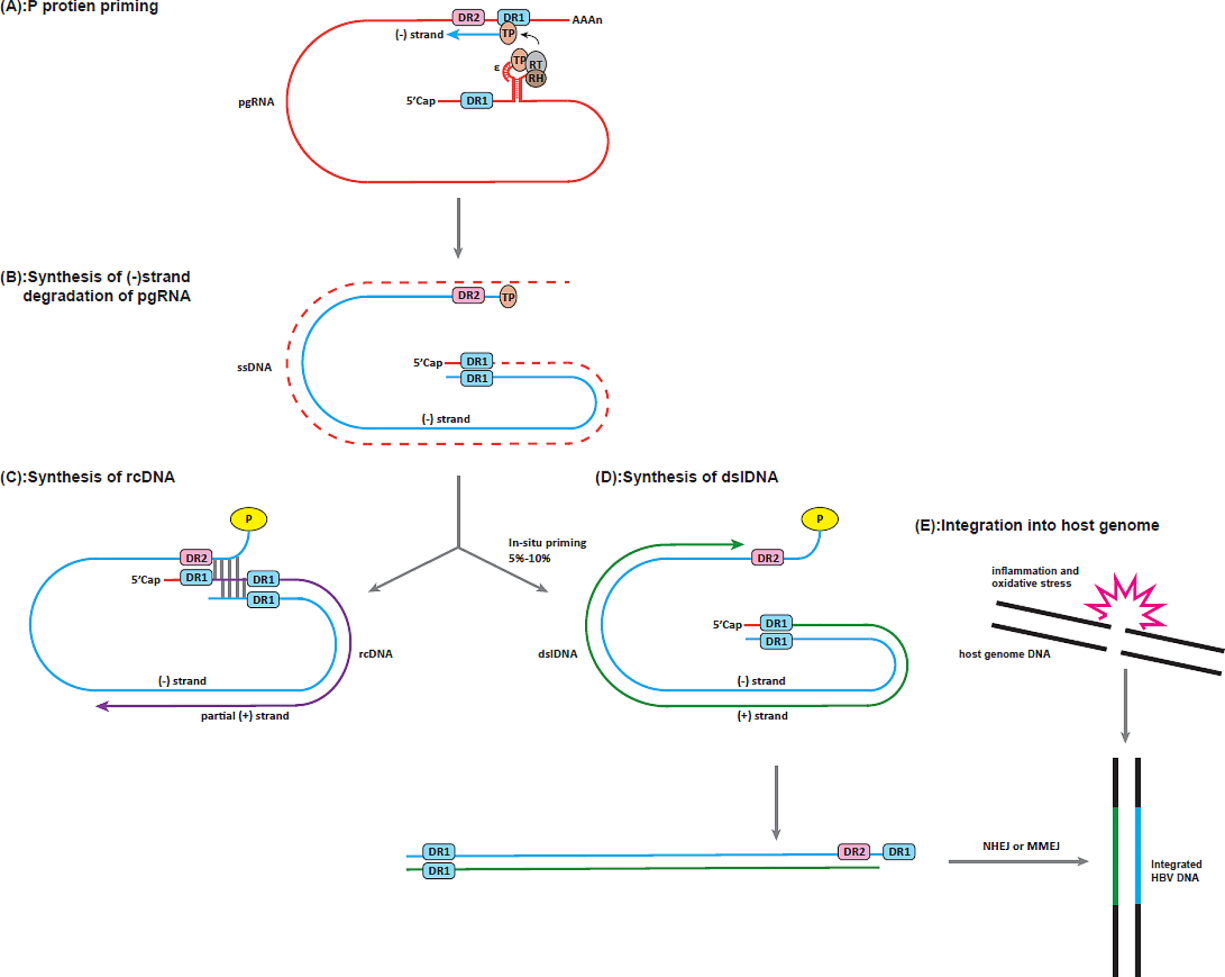
Schematic diagram about synthesis of dslDNA and HBV DNA integration. **(A)** ε stem-loop structure forms and P protein primes at the ε stem-loop structure to form P-ε ribonucleoprotein (RNP) complex. The terminal protein (TP) domain of the P protein binds with the first deoxyribonucleotide in ε stem-loop structure near the 5’cap of pgRNA. After the first four(TGAA) or three (GAA) nucleotides of the new minus(-) strand DNA generated in ε stem-loop structure, the DNA oligo then transferred to DR1 at the 3’end of pgRNA with TP and the synthesis of minus(-) strand starts.**(B)** pgRNA is degraded by RNase H domain of P protein while the minus(-) strand is synthesizing. **(C)** The DNA oligomer binds to the direct repeat 2 (DR2) region of the newly synthesized minus(-) strand DNA to guide the synthesis of plus(+) strand DNA, forming a partially circular rcDNA (90–95%). **(D)** The RNA primer directly initiated in situ without binding to the DR2 region (5–10%), dslDNA will be generated. **(E)** Inflammation and oxidative stress induce host genomic DNA double-stranded breaking, which provides breakpoints for integration through NHEJ or MMEJ

Since the initial discovery of HBV integration in the early 1980s [30, 31], several hypotheses have emerged to shed light on the mechanisms underlying this integration process [32, 33]. The dslDNA provides HBV DNA fragments that can integrate into the host genome. Upon entering the nucleus, dslDNA is inserted randomly into hepatocyte chromosomes through DNA repair pathways [23]. The oxidative damage caused by hepatitis can induce DNA breakages in the host genome, creating breakpoints for the integration process [34–38]. Considering the limited homologous sequences between viral DNA and the human genome, the most likely mechanisms for HBV integration are non-homologous end joining (NHEJ) and microhomology-mediated end joining (MMEJ) DNA repair pathways [38, 39]. These pathways facilitate the joining of DNA ends during the repair process, allowing the integration of HBV DNA fragments into the host genome. In the NHEJ pathway, DNA breaks lacking significant homology undergo modification and subsequent ligation, leading to the generation of deletions or insertions [40]. On the other hand, MMEJ pathway is a distinct mechanism for end joining that operates separately from NHEJ [41, 42]. MMEJ relies on the presence of microhomology and utilizes longer stretches of microhomology (5–25 bp) compared to NHEJ [41]. Furthermore, the dslDNA can undergo circularization through the NHEJ DNA repair pathway. However, this process can lead to the formation of non-functional molecules due to the error-prone nature of NHEJ (Fig. 2) [43].

The breakpoints of HBV integration in the viral genome display several distinctive characteristics: (1) Integration often takes place near the DR1 or DR2 sites. (2) Integrated HBV fragments show a range of sizes, varying from 28 bp to 3215 bp. Long integration fragments(> 2000 bp) are observed more frequently than short ones. (3) It is common to observe small deletions within viral sequences at the joining site [16, 38, 44–47]. These characteristics highlight the specific patterns and variations in HBV integration events within the viral genome. Previous studies have shown that HBV has a preference for integrating into genic regions such as exons, introns, and promoters, as well as gene-rich areas [48]. Notably, certain genes such as *hTERT*,* MLL4*, and *CCNE1* have been frequently identified as targets of HBV integration [44, 48–52]. This biased selection of integration sites has been observed in both tumor and adjacent tissues with a higher frequency of integration occurring in tumor tissues compared to non-tumor tissues [44, 53, 54]. These findings highlight the specific genomic locations where HBV integration tends to occur and suggest its potential impact on specific genes in both tumor and non-tumor tissues.

### HBV integration occurs early in infection

The occurrence of integration during the early stages of HBV infection has been supported by multiple studies, which aligns with experimental evidence from cell infection models and animal liver infection models. For instance, in ducklings experimentally infected with the avian hepadnavirus duck hepatitis B virus (DHBV), integrated HBV DNA was detected as early as 6 days post-infection [55]. Similarly, in the woodchuck infection model, integration of woodchuck hepatitis virus (WHV) was observed within 1–3 h post-infection, indicating immediate genomic integration of WHV DNA into hepatocytes upon natural viral invasion [45, 56]. These findings highlight the early occurrence of HBV integration and provide valuable insights into the dynamics of viral integration in different infection models. Moreover, numerous investigations utilizing primary human hepatocytes (PHH), HepaRG-NTCP, HepG2-NTCP, and Huh7-NTCP cells have consistently demonstrated rapid viral integration after infection [35]. Furuta et al. conducted a study using a chimeric mouse model consisting of human hepatocytes infected with HBV, where they found that HBV integration could occur between 23 and 49 days post-infection through MMEJ, primarily within mitochondrial DNA [57]. Furthermore, the occurrence of integration in acute hepatitis B also suggests its early onset following infection [58]. Taken together, these findings strongly indicate that integration may take place within the host genome during the initial stages of hepadnaviral infection. Considering that HBV DNA integration predominantly occurs during viral replication, it becomes crucial to hinder replication at the early stage of infection in order to prevent integration.

### The integration of HBV DNA and Hepatitis promotes each other

Liver damage caused by HBV infection is characterized by persistent necrotizing inflammation accompanied by immune regulation [59]. Integrations, as an early event in HBV infection, are closely associated with ongoing immune-mediated inflammatory responses. The oxidative damage to hepatocellular DNA acts as breakpoints for dslDNA integration [60]. Multiple studies consistently report a positive correlation between the extent of hepadnavirus integration and oxidative damage [61, 62]. From another perspective, HBV-specific cytotoxic T lymphocytes (CTLs) selectively target and eliminate hepatocytes replicating HBV, leading to the preferential clonal expansion of HBV DNA-integrated hepatocytes that may evade the immune response mediated by HBV-specific CTLs [47]. Moreover, the integration of HBV DNA can trigger an inflammatory response. Integrated HBV DNA is considered a potential source of HBsAg, which is derived from both a 2.1Kb transcript and mRNA transcribed from integrated HBV DNA. The presence of HBsAg plays a crucial role in the pathogenesis of hepatitis [63]. It is widely recognized that elevated production of HBsAg contributes to T cell exhaustion, resulting in restricted or impaired T cell responses and even the elimination of T cells recognizing specific epitopes [64, 65]. Additionally, CD205 has recently been identified as a pivotal receptor involved in the capture of CpG-oligodeoxynucleotides in vivo. The enhanced expression of CD205 on Kupffer cells in HBsAg-transgenic mice may be attributed to mild inflammation associated with HBsAg [66, 67]. In previous consensus, cccDNA has been acknowledged as the primary transcriptional template for HBsAg production [68, 69]. This hypothesis is further supported by evidence documented in chimpanzees with chronic HBV infection [70]. A dynamic observation using liver biopsy specimens from CHB patients revealed that individuals with HBV S gene integration experienced a slower decline in serum HBsAg levels compared to those without such integration following prolonged therapy [71]. This finding highlights the diverse origin of HBsAg. Researchers from Switzerland, based on liver biopsies obtained from HBe(-) patients, discovered that transcriptionally active integrated HBV DNA can autonomously generate HBsAg without relying on HBV replication [72]. This result may explain why serum HBsAg level is much less correlation with HBV DNA in HBe(-) patients with a very low HBV replication state.

The interaction between HBV DNA integration and hepatitis is complex, as they mutually reinforce each other through immune responses starting from the early stages of HBV infection, ultimately leading to the development of HCC.

## Examination and research models for HBV integration

With the progress of high-throughput sequencing technologies, different strategies have been implemented to enable a more precise investigation into the implications of the integration process. These strategies aid in the detection of integrated viral DNA within the host genome. The unique features of each strategy are summarized in Table 1.

### Examination methods based on DNA hybridization

The integration of HBV was initially detected in HCC patient tissue and the PLC/PRF/5 cell line in 1980 through Southern Blot hybridization using HBV as a probe^31^. It was observed that most integration events took place at the nicked cohesive end region of HBV DNA. Moreover, Northern blot analysis revealed the presence of specific transcripts of HBsAg even in the absence of HBcAg [73]. Following these discoveries, the Southern Blot hybridization technique was employed to detect viral integration within the host cell genome [2, 74]. Subsequent investigations progressively unveiled the integration of HBV DNA in liver tissues of patients with HBV-related conditions such as HCC, acute HBV hepatitis, chronic HBV infection, and HBV-related liver cirrhosis [2]. These findings highlight use of Southern Blot hybridization as a valuable tool in studying viral integration. In addition to this approach, in situ hybridization based on the same principle as Southern Blot hybridization was utilized to identify the chromosomal sites of HBV DNA integration [75]. Subsequently, Fluorescence In Situ Hybridization (FISH) emerged as a more sensitive and specific method for detecting integrated HBV DNA, replacing the previous techniques [76, 77].

### Examination methods based on PCR amplification

Recombinant plasmid vectors were utilized for the direct cloning of virus-cell junctions, allowing for a comprehensive examination of integrated HBV DNA fragments [78]. However, the presence of diverse virus-cell junctions poses significant challenges in achieving accurate and sensitive detection of HBV integration.

**Table 1:** Detection methods of HBV DNA integration

Technique	Suitable uses	Advantages	Limitations	References
Southern blot hybridization and FISH	Detection of the existence of HBV DNA integration in highly amplified clonal hepatocytes.	Low cost.	No sequence information. Dependent on restriction enzyme sites. Low sensitivity. Possibility of radioactivity.	[31, 79, 80]
Alu-PCR	Sequencing the detected integration in highly amplified clonal hepatocytes.	Low cost. Detection of integrated HBV DNA and adjacent cellular DNA sequence.	Detection of HBV integration near Alu sequences. Dependent on Alu sequences. No integration quantification.	[52, 81, 82, 83]
Inverse nested PCR	Detection of HBV DNA integrations and virus–cell junctions.	Quantification of integration. High selectivity and sensitivity. Low cost.	Dependent on restriction enzyme sites for detection of virus–host DNA junction. Detection of integrations that occur between nucleotides ~ 1650 and ~ 1850 of viral genome.	[16, 84, 85, 86]
Direct cloning and Sanger sequencing	Detection of HBV DNA integration in liver samples with highly expanded hepatocyte clones.	Detection of integrated HBV DNA and adjacent cellular DNA sequence.	Low throughput.	[44, 87]
Whole genome sequencing (WGS)	Sensitive and comprehensive in the identification of viral integrates across the human genome.	Full genome coverage.	High cost. Low depth.	[88, 89]
Whole-exome sequencing	Detection of HBV integration in coding regions.	Greater depth than WGS.	Only detection of coding region.	[90, 91]
Capture-enriched next generation sequencing	Targeted enrichment of integrated HBV DNA and sequencing.	Cost-effective compared with WGS. Quicker and less laborious than PCR-based methods High-throughput.	Lower sensitivity and specificity than PCR-based methods.	[92, 93, 94]
RNA sequencing	Detection the integrations in transcriptome.	Greater depth than WGS.	Only detection of expressed coding regions. Biased towards more highly expressed genes.	[88, 91, 94]

The detection of virus-cell junctions has been made possible through the development of various PCR-based strategies, including the Arthrobacter luteus-PCR(Alu-PCR) [95]. Alu elements, which are short interspersed nuclear elements (SINEs), are widely distributed throughout primate genomes and can be found in approximately 1,000,000 copies per human genome [96]. By utilizing a combination of HBV and Alu repetitive element primers, it is possible to amplify and sequence fragments of virus-cell DNA junctions [71, 97, 98]. However, Alu-PCR has limitations in detecting HBV integrated fragments that are located far from the Alu repeat sequence or accurately quantifying the integration junctions.

In addition to Alu-PCR, another technique called inverse nested PCR (invPCR) can be employed for amplifying virus-cell DNA junctions. This method provides an alternative approach to detect and analyze these junction fragments. In 1995, Gong et al. successfully detected DR-related integrations of wild-type DHBV in LMH-D2 cells using inv PCR, which introduced a novel protocol for detecting and characterizing integrations of DHBV derived from episomal viral DNAs [99]. This strategy was primarily designed to selectively amplify virus-cell DNA junctions near the DR sequences, as these DR sequences are recognized as preferred integration sites for hepadnaviral DNA [23, 45, 55, 99, 100]. To detect integrations in or near hypothetical sites, high-molecular-weight liver DNA was cleaved by restriction endonucleases specifically targeting and cleaving HBV DNA and host DNA at unknown sites. Subsequently, the DNA was circularized using T4 DNA ligase and further cleaved by another restriction endonuclease, resulting in the generation of linear strands. Within these strands, the viral-cellular DNA junctions were located internally, with viral fragments present at both termini. These fragments were then amplified through nested PCR utilizing virus-specific primers [16, 84]. This technique has been widely employed for the detection of integrated HBV DNA due to its high sensitivity and specificity [101–103]. However, it is important to note that this method can only detect DNA sequences in close proximity to the junctions and is heavily reliant on restriction endonucleases [47].

### Examination methods based on high-throughput sequencing technology

Whole-genome sequencing (WGS) and whole-exome sequencing (WES) are two widely used next-generation sequencing (NGS) methods that have found extensive applications in various areas of virology research. NGS offers several advantages, including the elimination of the need for prior viral DNA information and improved sensitivity in detection. WGS allows comprehensive coverage of host genomes, enabling the identification of viral sequences [104]. On the other hand, WES provides greater depth than WGS Nanopore sequencing, but it focuses solely on coding regions ^91^. However, deep sequencing with significant insertions or deletions remains challenging due to the intrinsic error-prone nature and limited length of the generated sequence reads [105]. In recent years, the field of third-generation sequencing technology has witnessed a remarkable advancement, offering inherent advantages in exploring complex genomic rearrangements [106]. This technology allows the generation of complete HBV genomes in a single sequencing read, facilitating the investigation of intricate and diverse distribution patterns of rapidly mutating viral genomes [107]. By combining third-generation sequencing with the analysis of biological information, a deeper understanding of HBV integration can be achieved [106].

### Research models for HBV DNA integration

Comprehensive investigations into HBV integration face challenges due to the limited availability of human non-tumor liver tissues at all stages of HBV infection, especially compared to HCC tissues. Additionally, the scarcity of suitable models for studying HBV infection further hampers research on HBV integrations. To overcome these limitations, several in vitro studies have utilized PHH, HepaRG-NTCP, HepG2-NTCP, and Huh7-NTCP cells to investigate the mechanisms and timing of HBV DNA integration [35, 57, 77]. These cell-based models offer valuable insights into HBV integration. Furthermore, other hepadnavirus-infected animal models have also contributed significantly. For example, studies using ducklings infected with DHBV and woodchucks infected with WHV have provided important contributions to our understanding of HBV integration [45, 55, 56]. These animal models offer insights that complement the in vitro studies and enhance our overall understanding of HBV integration. Since HBV integration in both genomic DNA and RNA transcripts was observed in various cell lines including HepG2.2.15, HepAD38, PLC/PRF/5, DE19, MHCC97H, MHCC97L, MHCCLM3 cells as well as Huh1 and Hep3B cells, [88, 92, 93] therefore, HBV-related HCC cell lines could also be utilized as the cell model for HBV integration. Animal infection models, including chimpanzees, human liver chimeric mice, Tupaia, and hNTCP-expressing macaques, have been utilized to study HBV infection. These models demonstrate susceptibility to chronic HBV infection and can generate clonally expanded hepatocytes that contain integrated viral DNA [17, 108–110].

## HBV DNA integration induces HCC

**Fig. 3:**
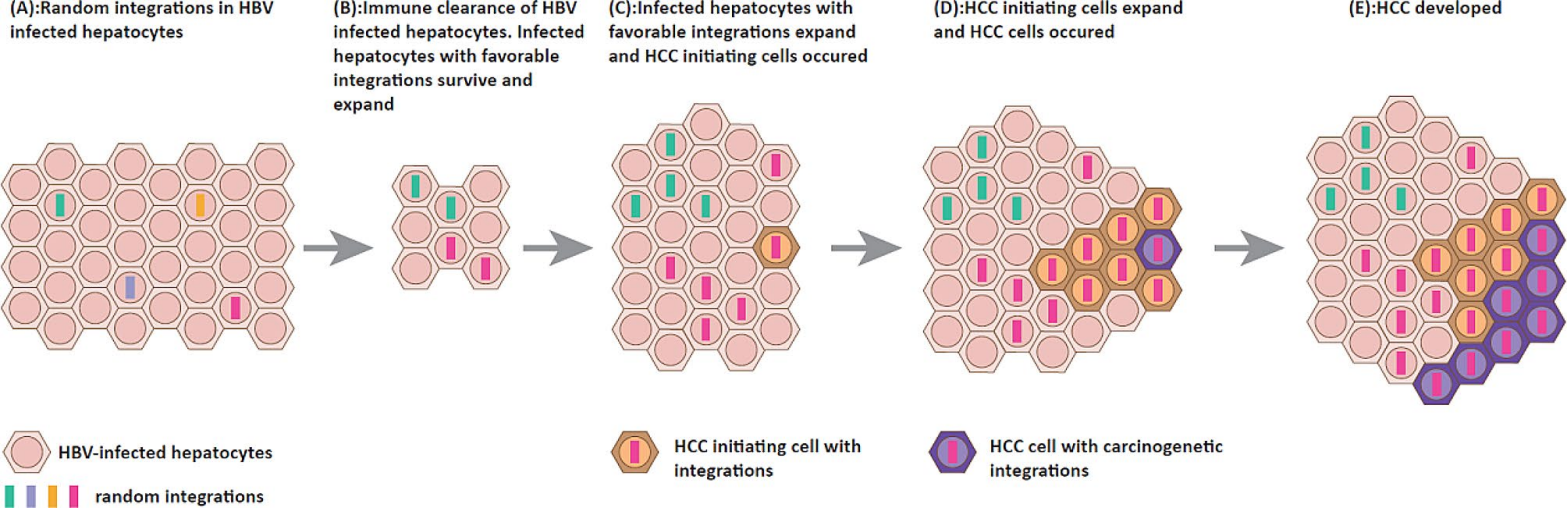
Schematic diagram of HBV DNA integration from chronic HBV infection to hepatocellular carcinoma. **(A)** Initially HBV DNA randomly integrates into host genome. **(B)** The infected hepatocytes are eliminated by host immune response. Infected hepatocytes with favorable integrations survive and clonally expand. **(C)** Hepatocytes with integrations expand. When integration happens near/into HCC-related genes, HCC initiating cells may occur. **(D)** HCC initiating cells expand and HCC cells with carcinogenetic integrations appear. **(E)** HCC cells with carcinogenetic integrations expand leading to the development of HCC

Previously, it was suggested that integrated HBV DNA had no discernible function due to its random distribution and lack of requirement in HBV replication. However, over the past decade, numerous studies have shown the significant impact of HBV DNA integrations on both HBV infection and carcinogenesis (Fig. 3) [5, 6, 32, 36, 46, 52, 101, 99, 111–121]. Therefore, conducting more research to understand the relationships between integration translocations in host genes, fragments of HBV genome, and carcinogenetic mechanism of integration are of great clinical significance [112]. The integration of HBV DNA into the host genome is an early event that precedes clonal tumor expansion [122, 123] and the presence of integration events indicates their potential role as precursors to tumor development in patients with chronic hepatitis and during the acute infection stage [35, 58, 124, 125]. HBV DNA integration primarily contributes to HCC through three mechanisms: (1) modulation of the expression or function of proto-oncogenes and tumor suppressor genes, (2) induction of chromosomal instability, and (3) expression of integrated mutant HBV proteins [54].

### HBV DNA integration modulates cancer-related genes

The integration of HBV in the human genome was observed to have a distinct distribution pattern in tumors compared to non-tumor tissues, with a tendency for enrichment around cancer driver genes [118]. The chromosomal locus 11q13.3 has a significant tendency to serve as a recurring site for HBV integration [126]. This specific genomic region contains crucial oncogenic driver genes, namely *CCND1* and *FGF19*, which are frequently amplified in HCC [127]. Additionally, the expression levels of cancer-associated genes, such as *hTERT*,* KMT2B*,* MLL4*, *CCNE1* and *PAK3*, were found to be up-regulated in tumor tissues compared to their corresponding normal counterparts [44, 128, 129]. Telomeres, which enhance telomerase activity, play a crucial role in maintaining genome stability. Additionally, the upregulation of *hTERT* has been extensively reported [130]. Furthermore, studies investigating *hTERT* integration sites have shown that HBV DNA integration at the *hTERT* promoter is pivotal in the overexpression of the *hTERT* gene [131, 132].

Integration events involving mitochondrial DNA have been identified in tissue samples obtained from both tumor and non-tumor areas of HCC patients. This new finding highlights mitochondrial DNA as a newly recognized target of HBV integration, causing mitochondrial instability and dysfunction. Consequently, this contributes to the development and progression of HCC [133]. In a recent study, the coexistence of two distinct HCC subtypes was observed in a patient with HBV infection, with no identical integration sites detected. This finding suggests that the multicentric occurrence of HCC may be attributed to diverse HBV DNA integration events [120].

### HBV DNA integration induces chromosomal instability

Chromosomal instability is a fundamental characteristic of human cancer, and it is closely associated with unfavorable prognosis, metastasis, and resistance to therapeutic interventions [134]. The breakpoints of HBV integration have been found to be correlated with an increased level of copy-number variation [44]. This observation highlights the potential contribution of HBV integration to the chromosomal instability observed in the HCC genome [38, 53].

One study suggests that HBV has a preferential integration site in the human genome, particularly fragile sites and CpG islands [38]. These are regions of the genome that are prone to rearrangements and genetic alterations, which can lead to the development of cancer. HBV integration into these regions can also lead to epigenetic instability, which can further contribute to the development of HCC [135]. Additionally, HBV integration events were observed to be enriched in the proximity of telomeres, which play a crucial role in maintaining genome stability. Dysfunction of telomeres can lead to extensive DNA rearrangements, deletions, and amplification, all of which are commonly associated with the development of cancer [136].

### HBV DNA integration expresses truncated HBV proteins

Truncated HBs and HBx proteins, derived from integration fragments of HBV DNA, are recognized as significant contributors to the development of HCC [137]. Truncated preS2/S sequences within hepatocytes, commonly observed in integrated HBV DNA, have been implicated in promoting HCC progression through multiple pathways [138–140]. The accumulation of truncated mutant HBsAg induces endoplasmic reticulum stress, leading to the generation of reactive oxygen species, oxidative stress, and DNA damage [141]. Moreover, the down-regulated expression of TGFBI induced by truncated HBsAg in the TGF-β/Smad signaling pathway also contributes to carcinogenesis [142]. Additionally, the truncated S protein impedes the G1/S phase cell cycle checkpoint by suppressing the expression of the p53-p21 axis [143]. Multiple truncated HBx proteins, particularly those with C-terminal truncation (ct-HBx), have been identified to exert diverse functions in HCC, including the induction of stem cell-like characteristics, inhibition of apoptosis, and promotion of HCC invasion and metastasis [144–150].

The incidence of HCC is significantly higher in males compared to females, with a ratio of approximately 4:1 but the reasons for the gender bias are unclear. Some certain integration sites of HBV can be identified as human somatic risk loci for HBV integration (VIMs). The enriched transcription factors in VIMs are involved in DNA repair and the androgen receptor (AR) signaling pathway. There are significant interactions between the AR pathway and the complement system. These interactions, along with the X-linked ZXDB regulon that includes albumin (ALB), may contribute to the male predominance observed in HCC [151]. However, additional research is required to confirm the association between HBV integration and male predominance in HCC. Furthermore, studying the underlying mechanisms of integrated HBV DNA in promoting HCC can aid in the development of more targeted therapeutic strategies for HCC and provide novel biomarkers for monitoring its occurrence.

## The quest for non-invasive biomarkers in HBV DNA integration during HCC development

Until now, the quantification of integrations has predominantly been conducted in liver tissues. However, liver biopsy is an invasive procedure associated with inherent risks. Alternative biomarkers such as serum levels of HBV core-related antigen (HBcrAg) and HBV RNA may serve as indicators of transcriptional activity specific to cccDNA, as they are expected to be exclusively generated from cccDNA rather than integrated HBV DNA due to the absence of a promoter that initiates core RNA transcription [22, 152–154]. Therefore, further investigation is needed to identify specific serum biomarkers for HBV DNA integration. Cell-free DNA (cfDNA) is an emerging noninvasive blood biomarker that is used to assess tumor progression, evaluate prognosis, diagnose diseases, and monitor response to treatment [155]. Recent studies have reported the detection of HBV integration in circulating cfDNA from both HCC and liver cirrhosis patients’ plasma [89]. Since cfDNA primarily originates from dying tumor cells, the release of cfDNAs from non-HCC liver tissues is considerably lower compared to HCC liver tissues [156]. As a result, cfDNA is more suitable for monitoring HBV integration in HCC development. In a study on the early recurrence of HCC after surgical resection, researchers found that plasma virus-host chimera DNA (vh-DNA) could serve as a biomarker for detecting residual tumor cells and predicting recurrence [94]. Detecting sequence-unknown vh-DNA directly from cfDNA requires a sensitive NGS approach with a standardized workflow and appropriate cutoff values, along with a population study to ensure sensitivity and specificity, incorporating known tumor-related somatic mutations [157].

## The importance of early intervention

HBV DNA integration has the potential to generate a portion of HBsAg and contribute to HCC development, making early intervention for HBV infection crucial. While NAs may not eradicate integrated HBV DNA, initiating treatment at an early stage can reduce the occurrence of integrations, potentially reducing oncogenic mutations. The continuous suppression of the virus through effective treatment significantly lowers the risk of oncogenic mutations. Functional cure, achieved through sustained virus suppression, greatly diminishes the likelihood of carcinogenic mutations. The elimination of cccDNA, the viral reservoir in hepatocytes, is essential in preventing HBV reactivation and relapse. The ultimate objective in managing HBV infection is to achieve a sterilizing cure, which involves the complete eradication of cccDNA and integrated HBV DNA from the host genome. Strategies aimed at accomplishing this goal include utilizing antiviral agents that specifically target and eliminate integrated HBV DNA from the host cells.

The slow decline, or no decline of serum HBsAg levels during NAs treatment may be due to the ongoing production of HBsAg from integrated HBV DNA, particularly in HBeAg-negative patients [158]. Recent studies have confirmed the presence of integrant-derived RNAs (id-RNAs) and 5’-human-HBV-3’ transcripts originating from integrated HBV DNA in serum [159]. Initiating treatment at an early stage may enhance the likelihood of achieving a functional cure by reducing HBV DNA integration. Additionally, quantifying integrations in these patients can help identify factors that contribute to the slow clearance of HBsAg.

## Exploring strategies for HBV DNA integration inhibition and elimination: current progress and future directions

The integration of HBV DNA can contribute to both neoplasia and a portion of HBsAg production. The elimination of integrated HBV DNA is also regarded as a critical measure for achieving complete eradication of HBV [1]. Inhibiting HBV DNA integration at an early stage holds immense importance and has consistently garnered significant attention from researchers in this field [160, 161]. The efficacies of current strategies to eliminate HBV DNA integration are concluded in Table 2.

**Table 2:** Efficacies of current strategies for HBV DNA integration inhibition and elimination

Treatment Strategies	Mechanism of antiviral treatment	Effect on integrated DNA	Advantages	Limitations	References
NAs	Inhibit HBV DNA polymerase, reducing viral replication.	Indirect effect: Suppress further integration events.	Effective in suppressing HBV replication. Gradually reduce the frequency of HBV integration events.	Do not eliminate cccDNA or integrated DNA. Risk of drug resistance over prolonged use.	[162–169]
IFN-α	Enhance the host immune response. Reduce the synthesis of pgRNA.	Indirect effect: Reduce new integration events.	Induce a functional cure in a minority of patients. Reduce the synthesis of pgRNA.	Limited effect on eliminating integrated HBV DNA. Associated with significant side effects and variable patient response.	[82, 169–171]
CRISPR/Cas9	Use guide RNAs to target and cleave specific HBV DNA sequences, including those within integrated DNA.	Direct effect: Specifically target and cleave integrated HBV DNA.	Potential to eliminate both cccDNA and integrated HBV DNA.	Risk of off-target effects and genome instability. Limited clinical application due to safety and delivery challenges.	[172–177]
RNAi	Use siRNAs to degrade viral mRNA, reducing the production of viral proteins and replication intermediates.	Indirect effect: Lowers the levels of viral mRNA and proteins.	Downregulate viral mRNA, reducing viral proteins, RNA, and DNA. Can lead to a reduction in integrated HBV DNA.	Do not directly affect cccDNA. Long-term efficacy and safety remain uncertain.	[70, 172, 178–183]
ZFNs and TALENs	Engineered nucleases that can be designed to target specific DNA sequences, including those within HBV integrations.	Direct effect: Induce double strand breaks at specific sites in the integrated HBV DNA.	Show potential in manipulating HBV cccDNA in cellular models. May help reduce integration events.	Early-stage research with limited clinical data. Potential off-target effects and technical complexity.	[184–191]

The commonly used treatment, such as NAs, has been recognized for its efficacy in inhibiting the production of integrated HBV DNA [71]. NAs are effective in suppressing HBV replication, thereby reducing the generation of integrated viral DNA resulting from viral replication. After entecavir (ETV) treatment, the pattern of HBV integration appears to be more random and irregular, potentially contributing to a decreased risk of HCC [162]. In a recent study, researchers procured liver tissue specimens from individuals diagnosed with chronic hepatitis B before the initiation of NAs treatment. Subsequently, they obtained liver tissue samples from the same individuals after five and ten years of continuous NAs treatment. This longitudinal analysis revealed a gradual reduction in the frequency of HBV integration events within the liver tissue over the specified treatment durations [167]. A plausible mechanism underlying this phenomenon is that NAs effectively suppress viral replication, while concomitant normal hepatocyte regeneration results in the gradual dilution of the frequency of viral integration events. However, it is important to note that NAs do not have any effect on eliminating cccDNA or integrated DNA [79, 168].

In comparison to NAs treatment, therapies that target innate immunity, such as IFN-α, are more likely to possess the potency to eliminate cccDNA [192]. This therapeutic approach has shown success in inducing a functional cure among a minority of patients with CHB in clinical settings [193]. Reports indicate that patients who are functionally cured and exhibit intrahepatic HBsAg possess higher levels of integrated HBV DNA than those without intrahepatic HBsAg. Interestingly, a certain subset of these patients maintains transcriptional activity of the integrated viral DNA [170, 171]. Utilizing spatial transcriptome sequencing, it was found that transcriptionally active HBV integration is relatively low in patients who have cleared HBsAg. In addition, there’s a close correlation between the level of intrahepatic cccDNA and virus integration events [194]. IFN-α has been shown to indirectly reduce the synthesis of pgRNA, which is vital for HBV DNA integration. Nevertheless, research is currently scant on whether IFN-α can completely eliminate integration. Thus, further exploration in this field is warranted.

The utilization of CRISPR/Cas9 has shown effectiveness in eliminating both HBV cccDNA and integrated HBV DNA [15, 176]. When selecting target sequences, it is important to optimize them to maximize the elimination of viral genes while minimizing potential damage to the human genome [195]. Following this principle, sequence design could focus on targeting the full-length 3,175-bp HBV DNA sequence [174]. Additionally, studies have explored targeting specific open reading frames of HBV, such as the S and X regions [175]. Both approaches hold promising potential for achieving a radical cure. However, the clinical application of CRISPR/Cas9 technology is currently limited due to factors like off-target cleavage and the risk of inducing genome instability when cutting integrated HBV DNA. A recently devised technique, involving the concurrent administration of Cas9 mRNA and guide RNAs, demonstrates its efficacy in modifying HBV integration DNA in mouse and tree shrew models, exhibiting a notable absence of liver enzyme elevation and minimal off-target effects [177]. A separate study put forth the hypothesis that pre-existing viral integrations within clonal HBV-infected hepatocytes could be eliminated during liver damage in patients with CHB. The researchers observed a negative correlation between the types and frequencies of breakpoints and the grade score for liver inflammation activity, providing support for this hypothesis [196].

It is intriguing to note that the majority of HBV transcripts show consistent termination sites within the viral genome, creating a unique opportunity to leverage RNA silencing mechanisms [70, 197]. RNA interference agents have emerged as a novel strategy for eradicating integrated DNA, with the potential to comprehensively influence the viral life cycle by downregulating all virus-generated mRNA [158]. One such agent, ARC-520, has shown promising results in reducing viral proteins, RNA, and DNA, leading to a surprising decrease in integrated HBV DNA in both chimpanzees and patients. However, it does not directly affect cccDNA [182].

Zinc-finger nucleases (ZFNs) or transcription activator-like effector nucleases (TALENs) have shown potential in manipulating HBV cccDNA in cellular models, which may help attenuate integration events [15, 161]. As our knowledge of cccDNA formation continues to grow, future therapeutic strategies could target nuclear enzymes, histones, and other essential components that play a crucial role in cccDNA generation [22].

## Conclusion

HBV integration refers to the insertion of DNA fragments derived from HBV into the human genome [119]. The integration of HBV DNA into the human genome has been extensively studied, revealing its confirmed carcinogenic potential in various experimental models. While integration events occur early during HBV infection, their exact role in the development of HCC is yet to be fully verified.

In the future, the establishment of comprehensive animal models that encapsulate the entire HBV infection process is pivotal. Such models will afford a more nuanced exploration of the ramifications of integrated HBV DNA on the hepatocytic transformation into a carcinogenic phenotype. Moreover, the development of pragmatic and cost-efficient methodologies for detecting integrations, coupled with the identification of pertinent serological markers denoting their presence, will significantly augment our capacity to appraise the potential for attaining a functional cure.

Furthermore, given that integrated HBV DNA contributes to the production of HBsAg and may impede the realization of a functional cure, prospective research should concentrate on discerning novel serological markers that more accurately signify the presence of integrations. This is particularly imperative in patients subjected to NAs treatment, where the absence of correlation between HBsAg levels and serum HBV DNA poses challenges in monitoring the efficacy of antiviral therapy.

Moreover, for the advancement of the field, the prioritization of clinical trials assessing the efficacy of diverse treatments in expediting the clearance of HBV integrations is essential. The exploration of innovative therapeutic modalities tailored specifically to target integrated HBV DNA will be instrumental in achieving comprehensive elimination. Thus, forthcoming research endeavors should be strategically oriented toward these pivotal domains to unravel the intricacies of HBV DNA integration and pave the way for more efficacious therapeutic interventions.

## Data Availability

No datasets were generated or analysed during the current study.

## Abbreviations

HBV: Hepatitis B virus
CHB: Chronic hepatitis B
HCC: Hepatocellular carcinoma
cccDNA: Covalently closed circular DNA
NAs: Nucleos(t)ide analogues
IFN: Interferon
HBsAg: Hepatitis surface antigen
HBc: Hepatitis B core protein
Pol: Polymerase
dsDNA: Double-stranded DNA
ORFs: Open reading frames
HBcAg: HBV core antigen
HBeAg: HBV e antigen
HBx: HBV X protein
rcDNA: Relaxed circular DNA
pgRNA: Pregenomic RNA
dslDNA: Double-stranded linear DNA
DR1: Direct repeat 1
DR2: Direct repeat 2
NHEJ: Non-homologous end joining
MMEJ: Microhomology-mediated end joining
RNP: Ribonucleoprotein
TP: Terminal protein
DHBV: Duck hepatitis B virus
WHV: Woodchuck hepatitis virus
PHH: Primary human hepatocytes
CTLs: Cytotoxic T lymphocytes
FISH: Fluorescence In Situ Hybridization
Alu-PCR: Arthrobacter luteus-PCR
SINEs: Short interspersed nuclear elements
invPCR: Inverse nested PCR
WGS: Whole-genome sequencing
WES: Whole-exome sequencing
NGS: Next-generation sequencing
ct-HBx: c-terminal truncation HBV X protein
AR: Androgen receptor
ALB: Albumin
HBcrAg: HBV core-related antigen
cfDNA: Cell-free DNA
id-RNAs: Integrant-derived RNAs
ETV: Entecavir
ZFNs: Zinc-finger nucleases
TALENs: Transcription activator-like effector nucleases
